# Laquinimod, a prototypic quinoline-3-carboxamide and aryl hydrocarbon receptor agonist, utilizes a CD155-mediated natural killer/dendritic cell interaction to suppress CNS autoimmunity

**DOI:** 10.1186/s12974-019-1437-0

**Published:** 2019-02-26

**Authors:** Martina Ott, Erika Avendaño-Guzmán, Evelyn Ullrich, Carolin Dreyer, Judith Strauss, Markus Harden, Margarete Schön, Michael P. Schön, Günter Bernhardt, Christine Stadelmann, Christiane Wegner, Wolfgang Brück, Stefan Nessler

**Affiliations:** 1 Institute of Neuropathology, University Medical Center Göttingen, Göttingen, Germany; 2 LOEWE Center for Cell and Gene Therapy, Goethe University, Frankfurt am Main, Germany; 3 Division of Stem Cell Transplantation and Immunology, Department for Children and Adolescents Medicine, Hospital of the Goethe University Frankfurt, Frankfurt am Main, Germany; 4 Institute for Multiple Sclerosis Research and Neuroimmunology, University Medical Center Göttingen, Göttingen, Germany; 5 Department of Medical Statistics, University Medical Center Göttingen, Göttingen, Germany; 6 Department of Dermatology, Venereology and Allergology, University Medical Center Göttingen, Göttingen, Germany; 7 Lower Saxony Institute of Occupational Dermatology, University Medical Center Göttingen and University of Osnabrück, Göttingen, Germany; 8 Institute of Immunology, Hannover Medical School, Carl-Neuberg-Straße 1, Gebäude I11 OE 5240, 30625 Hannover, Germany; 9 Present Address: Institute of Pathology, University Medical Center Göttingen, Göttingen, Germany

**Keywords:** Quinoline-3-carboxamide, Laquinimod, Natural killer cells, Aryl hydrocarbon receptor, Dendritic cells, DNAM-1/CD155 interactions, B16F10 melanoma cells, Experimental autoimmune encephalomyelitis, CNS autoimmunity

## Abstract

**Background:**

Quinoline-3-carboxamides, such as laquinimod, ameliorate CNS autoimmunity in patients and reduce tumor cell metastasis experimentally. Previous studies have focused on the immunomodulatory effect of laquinimod on myeloid cells. The data contained herein suggest that quinoline-3-carboxamides improve the immunomodulatory and anti-tumor effects of NK cells by upregulating the adhesion molecule DNAX accessory molecule-1 (DNAM-1).

**Methods:**

We explored how NK cell activation by laquinimod inhibits CNS autoimmunity in experimental autoimmune encephalomyelitis (EAE), the most utilized model of MS, and improves immunosurveillance of experimental lung melanoma metastasis. Functional manipulations included in vivo NK and DC depletion experiments and in vitro assays of NK cell function. Clinical, histological, and flow cytometric read-outs were assessed.

**Results:**

We demonstrate that laquinimod activates natural killer (NK) cells via the aryl hydrocarbon receptor and increases their DNAM-1 cell surface expression. This activation improves the cytotoxicity of NK cells against B16F10 melanoma cells and augments their immunoregulatory functions in EAE by interacting with CD155^+^ dendritic cells (DC). Noteworthy, the immunosuppressive effect of laquinimod-activated NK cells was due to decreasing MHC class II antigen presentation by DC and not by increasing DC killing.

**Conclusions:**

This study clarifies how DNAM-1 modifies the bidirectional crosstalk of NK cells with CD155^+^ DC, which can be exploited to suppress CNS autoimmunity and strengthen tumor surveillance.

**Electronic supplementary material:**

The online version of this article (10.1186/s12974-019-1437-0) contains supplementary material, which is available to authorized users.

## Background

Natural killer (NK) cells are large granular lymphocytes, which provide innate immune surveillance against virus infections and tumor cells [1]. Direct or indirect regulation of adaptive immune responses by NK cells may also affect the outcome of autoimmune diseases [2].

NK cells can rapidly kill tumor or pathogen-infected cells without prior sensitization and produce an array of cytokines and chemokines, which particularly influence dendritic cells (DCs). The bidirectional crosstalk between NK cells and DCs can lead to NK cell activation, DC maturation, or DC killing. Killing of tumor cells as well as most interactions between NK cells and DCs depend on cell contact and, therefore, require cell-to-cell adhesion [3]. DNAX accessory molecule 1 (DNAM-1, CD226) is such an adhesion and co-activating receptor on NK cells. It binds to the nectin family members CD155 and CD112 [4]. DNAM-1 thus facilitates NK cell interactions with tumor cells and CD155-expressing DCs. Recent studies have demonstrated the relevance of DNAM-1 for NK cell-mediated tumor cell elimination in the absence of NKG2D ligands, showing that DNAM-1 significantly contributed to the control of tumor growth and metastasis formation in experimental B16F10 melanoma models [5, 6]. Polymorphisms in the DNAM-1 gene, which decrease DNAM-1 protein expression on the cell surface, are risk factors for neoplastic diseases and have been linked to several autoimmune diseases, among them multiple sclerosis (MS) [7–9]. This suggests that diminished DNAM-1/CD155 interactions might not only reduce tumor cell killing but also impair the immunoregulatory capabilities of NK cells in controlling autoimmunity [10, 11].

Most studies in MS patients reported reduced numbers of circulating NK cells, which are functionally impaired with regard to cytotoxicity and interferon-gamma (IFNγ) production [12]. Evidence for a regulatory role of NK cells has been provided by studies with daclizumab, a humanized monoclonal antibody against the IL-2 receptor α-chain. In these studies, the expansion of peripheral and intrathecal CD56^bright^ NK cells correlated positively with the therapeutic response [13, 14]. In EAE, depletion of NK cells or diminished recruitment of NK cells to the CNS exacerbated EAE in most but not all studies [15–18].

The majority of data on regulatory NK cells in MS and EAE suggest that NK cells kill T cells directly or inhibit their proliferation [19, 20]. Gross et al. demonstrated that the reduced surface expression of DNAM-1 on NK cells of MS patients correlated with the impaired cytolysis of autologous, activated, CD155-expressing CD4 T cells [10]. The perforin-mediated killing of myelin oligodendrocyte glycoprotein (MOG)-specific T cells by NK cells was inhibited when the non-classical class 1 molecule Qa1 on T cells interacted with the NKG2A receptor on NK cells [21]. NK cells can also be demonstrated in the CSF of MS patients and in actively demyelinating MS lesions [10, 22], and regulatory functions of NK cells within the CNS have been postulated which require the interaction of NK cells with microglia cells [23].

Quinoline-3-carboxamide derivatives such as laquinimod, tasquinimod, or pasquinimod have shown immunomodulatory, anti-tumor, and anti-angiogenic effects in pre-clinical animal models [24–26]. So far, the modulation of myeloid cells was primarily held responsible for their immunomodulatory and anti-tumor effects [27–31]. Here, we provide evidence that the aryl hydrocarbon receptor-dependent activation of NK cells is relevant for the efficacy of laquinimod to suppress melanoma cell metastases and CNS autoimmunity. The NK cell-mediated inhibition of T cell proliferation is dependent on cell-to-cell contact and requires the interaction of DNAM-1 with CD155 on dendritic cells.

## Methods

### Animals

C57BL/6-Ahr^tm1.2Arte^ and Itgax-DTR/EGFP mice [32] were purchased from Taconic and the Jackson Laboratory, respectively. CD155-deficient mice were kindly provided by Prof. Bernhardt. Rag1^−/−^ [33], myelin oligodendrocyte glycoprotein (MOG)-specific T cell receptor (TCR) transgenic mice (also referred to as 2D2 mice [34]), MOG-specific Ig heavy-chain knock-in mice on a C57BL/6 background (also referred to as Th mice [35]), and C57BL/6 mice were bred at the animal facility of the University Medical Center Göttingen under SPF conditions. All animals were housed in a temperature-controlled environment with 12-h light/dark cycles and food and water ad libitum. All animal procedures were approved by the Lower Saxony Federal State Office for Consumer Protection and Food Safety (LAVES), Germany.

### EAE induction

For EAE induction, Th/+ animals were immunized s.c. with 50 μg MOG_35–55_ per animal emulsified in CFA substituted with 5 mg/ml H37Ra (DIFCO, Detroit, MI, USA). Four hundred nanograms of pertussis toxin (PTX) per animal (List Biological Laboratories, Campbell, CA, USA) were injected i.p. twice on day 0 and day 2 relative to immunization. EAE was induced in Rag1^−/−^ mice by the adoptive transfer of 10 Mio MOG-specific 2D2 T cell blasts i.p., and animals were immunized s.c. with 10 μg MOG_35–55_ in CFA the following day. EAE was scored daily as previously described [36].

### Cell depletion

NK cells were depleted in Th/+ mice or Rag1^−/−^ mice by i.p. injections of 300 μg aNK1.1 antibody (clone PK136) per animal, and control mice received i.p injections of 300 μg C1.18.4 per animal (both Bio X Cell, West Lebanon, NH, USA). Depletion started at days − 2 and − 1 relative to immunization in Th/+ mice or adoptive T cell transfer into Rag1^−/−^ mice and continued every other day thereafter. Depletion efficiency in the blood was evaluated by flow cytometry prior to MOG_35–55_ immunization. DCs were depleted in Itgax-DTR/EGFP mice by injection of 100 ng diphtheria toxin per animal (Sigma Aldrich) 24 h prior to laquinimod or vehicle treatment. DC depletion efficiency was analyzed by flow cytometry in the spleens at day 3.

### Laquinimod therapy

Mice were treated with the quinoline-3-carboxamide laquinimod or vehicle (H_2_O) by oral gavage on a daily basis. All EAE-induced animals received 25 mg/kg laquinimod starting at the day of EAE induction. Laquinimod (50 mg/kg) treatment was initiated in animals three days prior to the i.v. injection of 500,000 B16F10 melanoma cells (preventive treatment) or 11 days after the i.v. injection of 200,000 B16F10 melanoma cells (therapeutic treatment).

### T cell culture

Spleen cells from MOG TCR transgenic 2D2 mice were expanded in RPMI 1640 supplemented with 10% FCS, 50 IU/ml penicillin, 50 μg/ml streptomycin (P/S), 2 mM L-glutamine, 1 mM sodium pyruvate, and 50 μM β2-mercaptoethanol (complete medium) by plate-bound αCD3 (clone 145C11, 4 μg/ml) and freely available αCD28 (clone PV-1, 1 μg/ml, both Bio X Cell) antibodies in the presence of rm IL-12 (1 ng/ml, R&D) and rm IL-2 (2 ng/ml, R&D). Before the adoptive transfer into recipient Rag1^−/−^ mice, cells were restimulated using 30 Gy irradiated syngenic splenocytes and 20 μg/ml MOG_35–55_ for 3 days.

### B16F10 cells

B16F10 melanoma cells (ATCC® CRL-6475™) were previously subcloned from the C57BL/6J-derived melanoma cell line B16 and selected for their ability to form pulmonary tumor nodules [37]. Cells were maintained in complete medium and split at 50% confluence.

### Bone marrow-derived dendritic cells

For the generation of bone marrow-derived dendritic cells, femurs were flushed and single-cell suspensions were cultured with 25 ng/ml rm GM-CSF (PeproTech, Rocky Hill, USA) in complete medium for 7 days and then MACS sorted.

### In vitro NK cell assays

Splenocyte suspensions of laquinimod- or vehicle-treated mice were enriched for NK cells by magnetic-activated cell sorting (MACS™) using the untouched NK cell isolation kit (Miltenyi Biotec, Bergisch Gladbach, Germany) and further purified to a purity > 99% using a FACSAria II cell sorter (BD Biosciences). CD4^+^ T cells were purified from healthy 2D2 mice by MACS with the untouched CD4 T cell isolation kit (Miltenyi Biotec) and CFSE labeled according to the manufacturer’s instructions (CellTrace™, Life Technologies, Carlsbad, USA). Bone marrow-derived DCs (bm DCs) were MACS purified with CD11c microbeads (Miltenyi Biotec) on day 7.

Splenic NK cells from laquinimod- or vehicle-treated mice were MACS sorted with the untouched NK cell isolation kit, separated into CD27 and CD11b single-positive NK cells by cell sorting and cultured at different effector/target ratios with 5000 B16F10 melanoma cells in the presence of 5000 U/ml rh IL-2 for 48 h. The tumor-lytic capacity was assessed by the crystal violet assay.

MACS-purified bmDCs were stimulated with 50 ng/ml LPS (Sigma Aldrich, St. Louis, USA) overnight, washed carefully, and co-cultured with MACS-sorted, CFSE-labeled 2D2 T cells and purified NK cells derived from the spleens of laquinimod- or vehicle-treated animals. Fifty thousand DCs, 50,000 NK cells, and 100,000 2D2 T cells/well were cultured for 72 h with or without 20 μg/ml MOG_35–55_, and the CFSE profile was analyzed by flow cytometry. In some experiments, NK cells and DCs were separated by a transwell membrane (Corning, Corning, USA) or T cells were stimulated in the absence of DCs with plate-bound αCD3 (145-2C11, 4 μg/ml) and soluble αCD28 (PV-1, 1 μg/ml) antibodies.

Purified splenic NK cells from laquinimod- or vehicle-treated animals were stimulated overnight with 1 ng/ml IL-12 and 25 ng/ml IL-18 (both PeproTech, Rocky Hill, USA), and IFNγ concentrations in the supernatant were determined by ELISA (R&D, Minneapolis, USA). Alternatively, IFNγ was analyzed in purified NK cells by intracellular cytokine staining, which were stimulated with IL-12 and IL-18 for 18 h, the final 6 h in the presence of GolgiStop™ (BD Biosciences, Franklin Lakes, USA).

Untouched mouse NK cells purified by MACS and cell sorting were cultivated in RPMI 1640 supplemented with 10% FCS, P/S, 2 mM L-glutamine, 1 mM sodium pyruvate, 50 μM β2-mercaptoethanol, and 1000 U/ml rh IL-2 for 48 h in the presence of 10 μM laquinimod.

Untouched human NK cells were purified by MACS and cell sorting and cultured in RPMI 1640 supplemented with 10% FCS, 2 mM L-glutamine, 1 mM sodium pyruvate, 50 μM β2-mercaptoethanol, and 50 U/ml rh IL-2 (Novartis, Nuremberg, Germany) for 120 h in the presence of 15 μM laquinimod.

### In vivo tumor assay

For the induction of lung metastases, animals treated with laquinimod (50 mg/kg) or vehicle were i.v. transferred into the tail veins with single-cell suspensions of B16F10 cells. Lungs were obtained on day 10 or day 19, weighted, and immersion fixed in 4% PFA. The tumor nodules were counted on both sides of each lung lobe with the aid of a dissection microscope.

### Flow cytometry

Single-cell suspensions were incubated for 15 min in Fc-blocking buffer and stained with the following anti-mouse antibodies (if not stated otherwise, all from BioLegend, San Diego, USA, or eBioscience, San Diego, USA): αCD3 (145-2C11), αCD4 (RM4-5), αCD8 (53-6.7), αCD11b (M1/70), αCD11c (N418), αCD16 (R&D Systems, Wiesbaden, Germany, 275003), αCD19 (eBio1D3), αCD27 (LG.3A10), αCD40 (3/23), αCD45 (30-F11), αCD80 (16-10A1), αCD86 (GL-1), αCD112 (R&D, 829038), αCD155 (TX56), αAHR (4MEJJ), αDNAM-1 (10E5), αNKG2D (CX5), αTIGIT (1G9), αLy49C/F/I/H (14B11), αLy49C/I, αLy49D, αMHC class II (M5/114), αTRAIL (N2B2), αNK1.1 (PK-136), αNkp46 (29A1.4), and αIFNγ (XMG1.2).

Anti-human antibodies were as follows: αCD3 (SK7), αCD16 (3G8), αCD14 (Beckman Coulter, Brea, USA, RMO52), αCD19 (HIB19), αCD56 (BD, NCAM16.2), αCD69 (FN50), αCCR7 (TG8), αDNAM-1 (11A8), αNKG2D (1D11), αNKp30, αNKp46 (9E2), and αTIGIT (MBSA43).

For intracellular staining, cells were fixed after surface staining for 45 min and permeabilized for 30 min with permeabilization buffer/antibody using the BD intracellular staining kit.

All flow cytometry data were acquired on a BD FACSCanto™ II and analyzed with FlowJo Tree Star software.

### Statistical analysis

Statistics were calculated using GraphPad Prism 5.01. Data were tested for normal distribution with the help of the Kolmogorov-Smirnov test with Dallal-Wilkinson-Lillie for *p* value. To compare two experimental groups, unpaired *t* tests were used for parametric data and Mann-Whitney *U* tests for non-parametric data. To compare three or more groups, one-way ANOVA with Bonferroni or Dunnett’s post-test was performed for parametric data and the Kruskal-Wallis test with Dunn’s post-test was applied for non-parametric data. Survival analysis was calculated with the log-rank test. All statistical analyses of EAE scores in Rag1^−/−^ and Th/+ mice after NK cell depletion were performed using two-way ANOVA with Tukey’s multiple comparison test. Statistical significance was defined as *p* < 0.05. Data are presented as mean ± SEM, if not otherwise stated.

## Results

### Activation of NK cells by the quinoline-3-carboxamide laquinimod requires the aryl hydrocarbon receptor

**Fig. 1:**
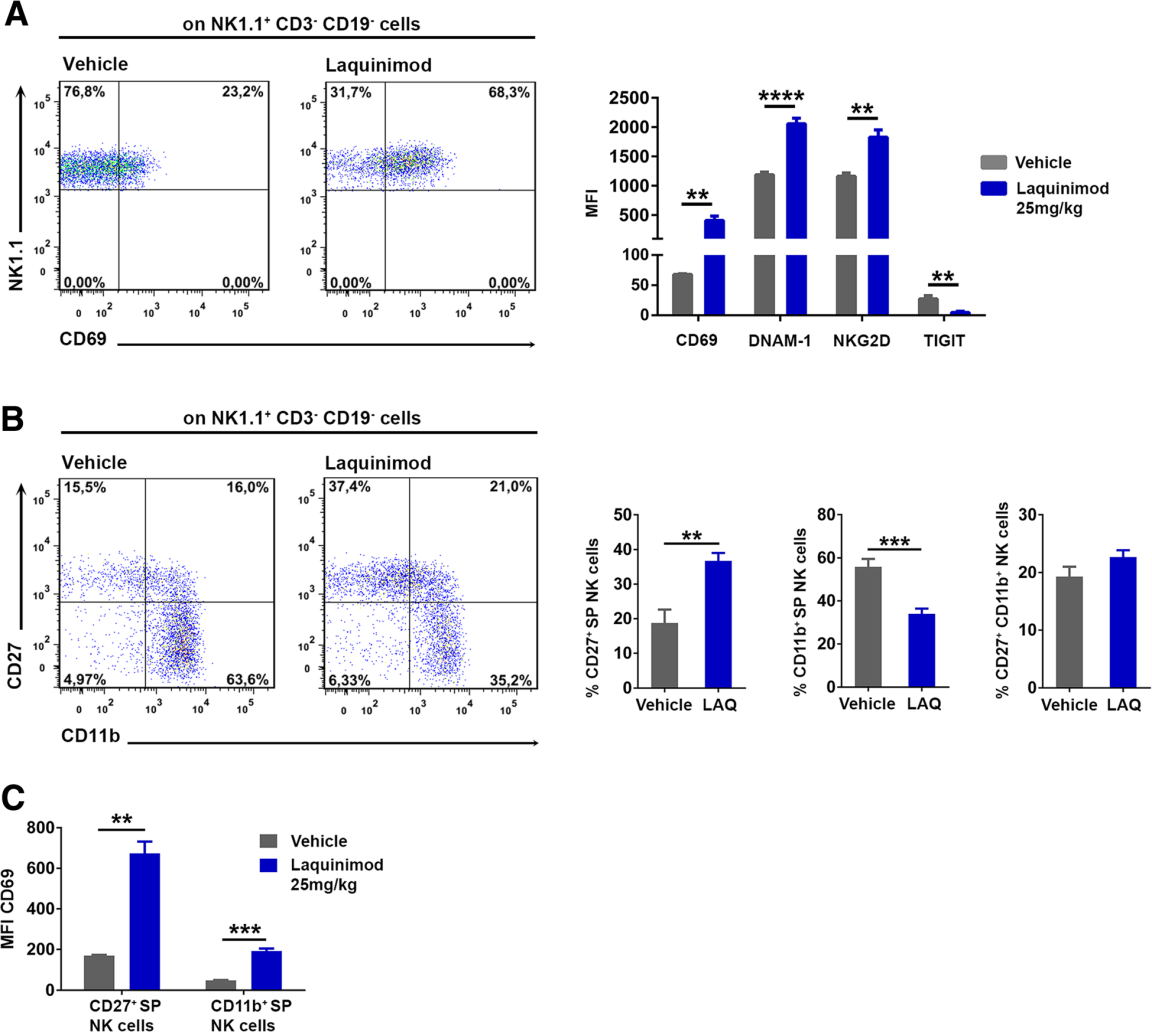
Laquinimod therapy activates NK cells. **a** Representative flow cytometry analysis of NK1.1/CD69 expression and quantification of activating and inhibitory NK cell receptors on day 11 after immunization with MOG_35–55_ and laquinimod (blue bars) or vehicle therapy (gray bars). Data are presented as mean ± S.E.M. and are representative of two independent experiments with five animals/group. **P < 0.01, ***P < 0.001, ****P < 0.0001, unpaired *t* test with Welch’s correction. **b** Representative flow cytometry analysis of splenic NK cell subsets defined by CD27/CD11b expression on day 11 after laquinimod or vehicle therapy of MOG_35–55_-immunized animals. Data are presented as mean ± S.E.M. and are pooled from three independent experiments with nine animals/group. **P < 0.01, ***P < 0.001, unpaired *t* test. **c** Quantification of CD69 expression by flow cytometry on NK cell subsets. Data are presented as mean ± S.E.M. and are representative of two independent experiments with five animals/group. **P < 0.01, ***P < 0.001 unpaired *t* test

Activation of NK cells is finely tuned by the integration of multiple activating and inhibitory signals delivered by many receptor-ligand interactions. We first analyzed whether daily oral treatment with laquinimod shifts the balance of activating versus inhibitory NK cell receptors in the spleens of MOG_35–55_-immunized C57BL/6 mice at day 11 after immunization. NK cells upregulated the early activation marker CD69 and the activating receptors NKG2D and DNAM-1 in response to laquinimod therapy (Fig. 1a). In contrast, animals treated with laquinimod downregulated the inhibitory NK cell receptor TIGIT (T cell immunoreceptor with Ig and ITIM domains) that binds to CD155 with higher affinity than DNAM-1 (Fig. 1a). DNAM-1 expression was downregulated, and TIGIT expression was unchanged in T cells by laquinimod treatment (Additional file 1: Figure S1A, B).

The immunoregulatory functions of human NK cells have been attributed primarily to the CD56^bright^ NK cell subpopulation, a surface marker not present in mouse NK cells. NK cell subpopulations in the mouse can be defined by CD27 and CD11b antibodies [38], and human CD56^bright^ NK cells correspond best to CD27 single-positive mouse NK cells. Laquinimod therapy significantly increased the percentage of CD27^+^ single-positive (SP) NK cells and decreased the percentage of CD11b^+^ SP NK cells (Fig. 1b), and both subsets were activated in response to laquinimod therapy (Fig. 1c).

**Fig. 2:**
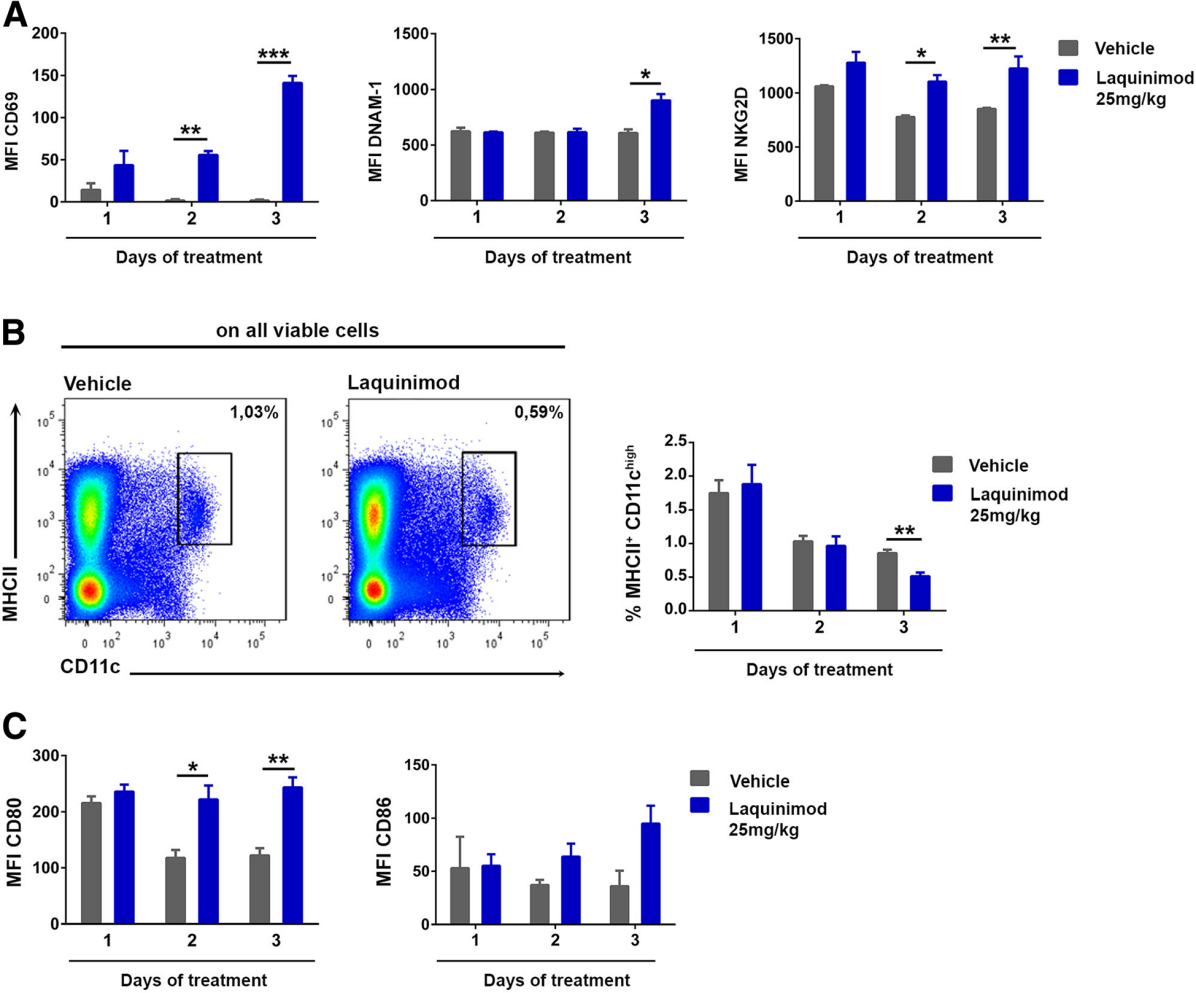
NK cells and DCs rapidly respond to laquinimod therapy. **a** Graphs show the mean fluorescence intensity (MFI) of activating NK cell markers as calculated from flow cytometry data at different time points. Cells were derived from laquinimod- or vehicle-treated MOG_35–55_-immunized animals. Data are presented as mean ± S.E.M. and are representative of four independent experiments with three animals per group and time point. *P < 0.05, **P < 0.01, ***P < 0.001, unpaired *t* test. **b** Representative flow cytometry analysis of MHC class II/CD11c expression in the spleens of laquinimod- or vehicle-treated animals. Frequency of MHC class II^+^ CD11c^high^ cells in the spleens of laquinimod- or vehicle-treated animals immunized with MOG_35–55_ as calculated from flow cytometry data at different time points. **c** MFI of CD80 and CD86 staining on splenic DCs of laquinimod- or vehicle-treated MOG_35–55_-immunized animals at different time points. Data are presented as mean ± S.E.M. and are representative of two independent experiments with three animals/group and time point. *P < 0.05, **P < 0.01, unpaired *t* test

**Fig. 3:**
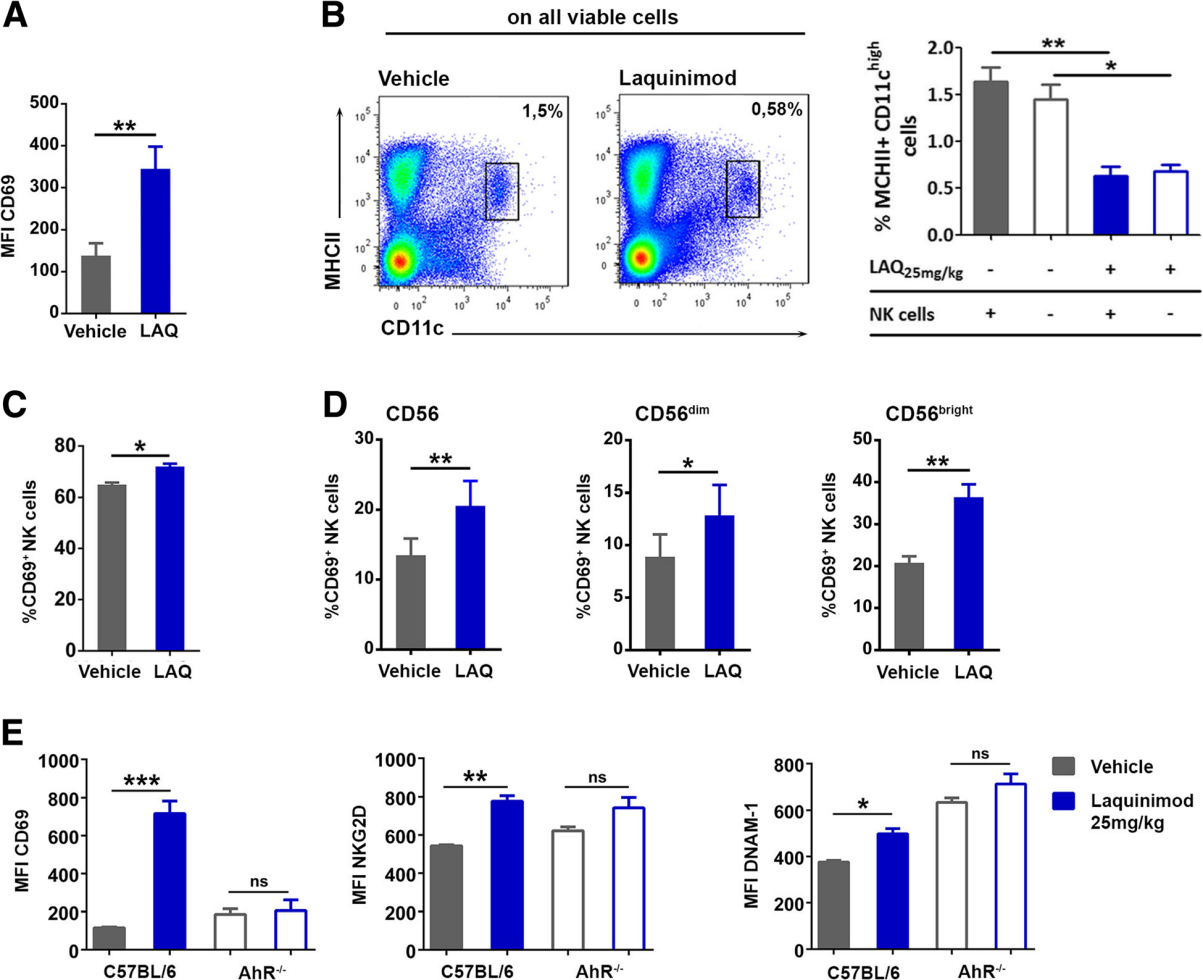
NK cells and DCs respond to laquinimod therapy independently. **a** Expression of CD69 (MFI) on splenic NK cells of Itgax-DTR mice treated for 3 days with laquinimod or vehicle. Itgax-DTR mice had been depleted of DCs 24 h prior to laquinimod therapy by 100 ng diphtheria toxin. Data are presented as mean ± S.E.M. and are representative of two independent experiments with six animals/group. **P < 0.01, unpaired *t* test. **b** Representative flow cytometry analysis of MHC class II/CD11c expression in NK cell-depleted spleens at day 11 post-MOG_35–55_ immunization. Frequency of MHC class II^+^ CD11c^high^ cells in the spleens of NK cell-competent (filled bars) and NK cell-depleted (hollow bars) mice treated with vehicle (gray) or laquinimod (blue) as assessed by flow cytometry. Data are presented as mean ± S.E.M. and are representative of two independent experiments with three animals/group. *P < 0.05, **P < 0.01, one-way ANOVA. **c** Frequency of CD69^+^ NK cells in purified splenic mouse NK cell cultures treated with 1000 U/ml IL-2 and 10 μM laquinimod or vehicle for 48 h. Data are presented as mean ± S.E.M. and are representative of three independent experiments. *P < 0.05, unpaired *t* test. **d** Frequency of CD69^+^ NK cells in sorted human NK cell cultures treated with 50 U/ml IL-2 and 15 μM laquinimod or vehicle for 120 h. Data are presented as mean ± S.E.M. and are representative of two independent experiments. *P < 0.05, **P < 0.01, unpaired *t* test. **e** CD69, NKG2D, and DNAM-1 expression (MFI) in naïve C57BL/6J or AhR^−/−^ mice treated for 11 days with 25 mg/kg laquinimod or vehicle. Data are presented as mean ± S.E.M. and are representative of two independent experiments with five animals per group. ***P < 0.001, two-way ANOVA

The activation of NK cells by laquinimod was detectable already at day 2 after treatment onset (Fig. 2a). As such, the NK cell response paralleled the changes observed in the DC compartment (Fig. 2b, c) and preceded the induction of Tregs (data not shown). The bidirectional crosstalk between DC and NK cells, which influences their activation status, is well established. Therefore, we evaluated if laquinimod activates NK cells in Itgax-DTR mice, which express the diphtheria toxin receptor (DTR) in CD11c^+^ cells, allowing the conditional depletion of DC. In reciprocal experiments, we depleted NK cells by anti-NK1.1 antibodies and analyzed the treatment effect of laquinimod on the DC compartment. Laquinimod treatment activated NK cells in animals with significantly reduced DC numbers (Fig. 3a, Additional file 2: Figure S2A) or in Rag1^−/−^ animals deficient of adaptive immune cells (data not shown) and reduced the frequency of DCs in NK cell-depleted mice (Fig. 3b, Additional file 2: Figure S2B). Furthermore, laquinimod activated highly purified mouse NK cells in vitro (Fig. 3c). To confirm the effects seen on murine NK cells, we treated purified human NK cells with laquinimod, which significantly activated both CD56^bright^ and CD56^dim^ human NK cell subsets (Fig. 3d).

The aryl hydrocarbon receptor (AhR), one of the bHLH PAS domain transcription factors, is present in NK cells (Additional file 3: Figure S3A). It was recently established as a regulator of NK cell activation and dendritic cell immunogenicity [39]. Furthermore, the therapeutic efficacy of laquinimod in EAE was recently shown to be dependent on the AhR [40]. Thus, we tested if laquinimod was capable of activating NK cells in AhR-deficient mice. Laquinimod treatment did neither activate NK cells (Fig. 3e, Additional file 3: Figure S3B) nor reduce the number of CD11c^high^ MHC class II^+^ DCs in AhR-deficient mice (Additional file 3: Figure S3C). In summary, we provide evidence that NK cells are activated directly by laquinimod in vivo and in vitro. The NK cell-activating properties of laquinimod are AhR-dependent.

### Laquinimod improves NK cell effector functions and reduces pulmonary B16F10 metastases

**Fig. 4:**
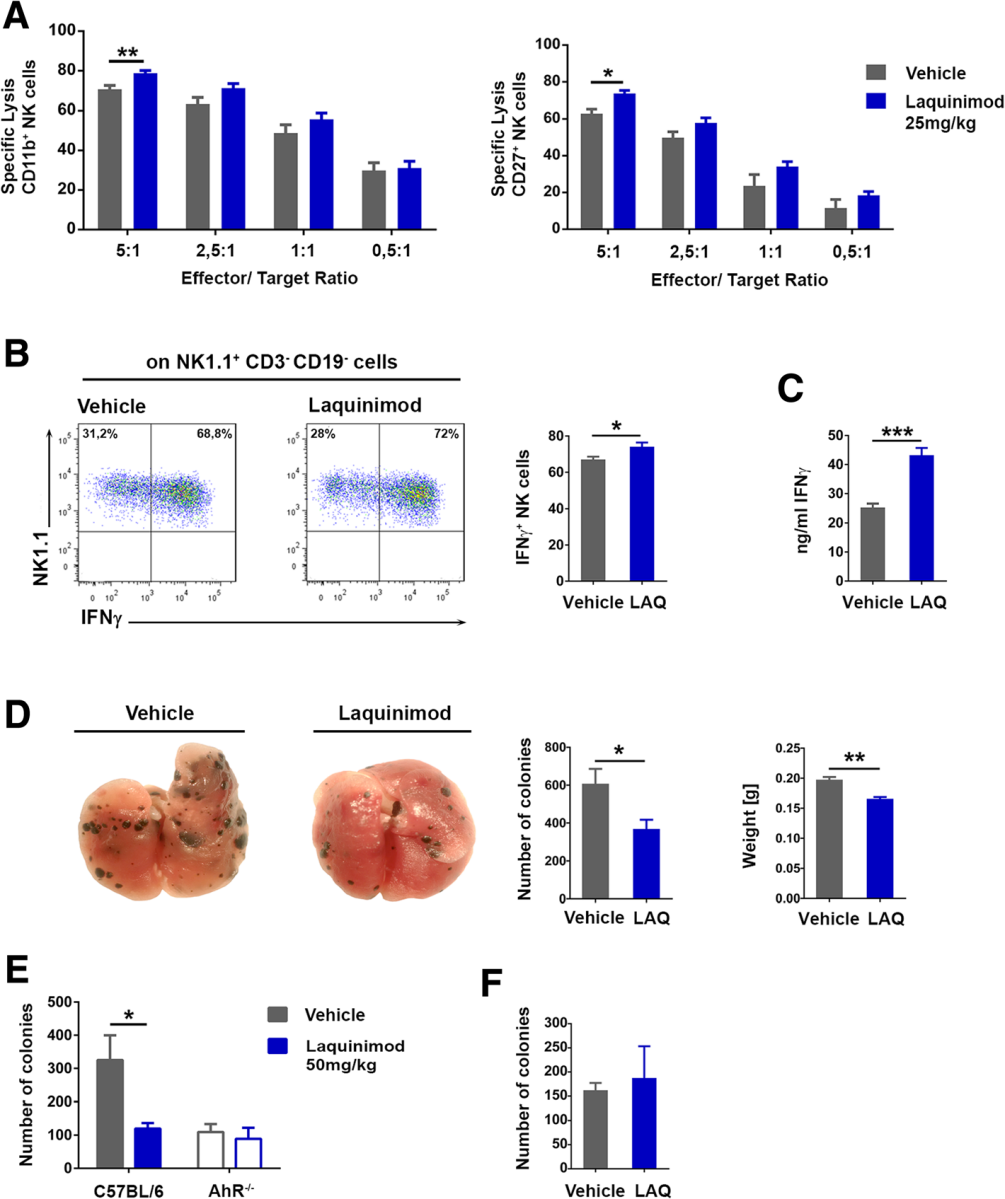
Laquinimod improves NK cell effector functions. **a** Killing of B16F10 cells (target) by ex vivo sorted splenic NK cell subsets (effector) from laquinimod- (25 mg/kg) or vehicle-treated mice as assessed by the crystal violet assay. Data are presented as mean ± S.E.M. and are representative of three independent experiments. **P < 0.01, unpaired *t* test. **b** Representative FACS plot showing the intracellular IFNγ content in splenic NK cells from laquinimod- or vehicle-treated mice 18 h after IL-12 (1 ng/ml) and IL-18 stimulation (25 ng/ml). Bar graph depicts mean ± S.E.M. pooled from two independent experiments (*n* = 8/ group). *P < 0.05, unpaired *t* test. **c** IFNγ concentrations in the supernatant of IL-12 and IL-18 stimulated splenic NK cells purified from laquinimod- or vehicle-treated mice. The graph depicts mean ± S.E.M. pooled from two independent experiments (*n* = 8/group), **P < 0.01, unpaired *t* test. **d** Metastatic burden in the lungs of preventively laquinimod- (50 mg/kg) or vehicle-treated mice 10 days after i.v. injection of B16F10 melanoma cells (5 × 10^5^) assessed by the quantification of lung nodules and lung weight. Data are presented as mean ± S.E.M. and are representative of four independent experiments. *P < 0.05, **P < 0.01, unpaired *t* test. **e** Metastatic burden in the lungs of preventively laquinimod- (50 mg/kg) or vehicle-treated C57BL/6 and AhR^−/−^ mice. Data present mean ± S.E.M. pooled from three independent experiments (12–21 animals/group). *P < 0.05, two-way ANOVA. **f** Metastatic burden in the lungs of laquinimod- (50 mg/kg) or vehicle-treated mice 19 days after i.v. injection of 2 × 10^5^ B16F10 melanoma cells if treatment was initiated 11 days after melanoma cell transfer

An increase in activating NK cell receptors, accompanied by a decrease in inhibitory NK cell receptors, is likely to translate into augmented NK cell effector functions. To confirm this assumption, we purified NK cells ex vivo from laquinimod-treated animals and analyzed whether they could kill B16F10 melanoma cells more effectively than NK cells from vehicle-treated controls. We also investigated whether they produced more cytokines in response to IL-15 and IL-18 stimulation compared to NK cells purified from vehicle-treated controls. Both CD27^+^ and CD11b^+^ NK cells from laquinimod-treated animals killed B16F10 melanoma cells more efficiently than CD27^+^ and CD11b^+^ NK cells from vehicle-treated controls (Fig. 4a). In addition, NK cells from laquinimod-treated animals also produced more IFNγ in response to IL-15 and IL-18 stimulation than NK cells purified from vehicle-treated controls (Fig. 4b, c).

DNAM-1 expression might identify anti-tumor NK cells [41]. DNAM-1^+^ NK cells are particularly relevant for the suppression of B16F10 melanoma cells [5], which do not display NKG2D ligands but express the DNAM-1 receptors CD155 and CD112 (Additional file 4: Figure S4A). We therefore investigated whether laquinimod treatment leads to a better control of melanoma metastasis formation and growth in vivo. In a series of experiments, C57BL/6 mice received laquinimod or vehicle daily, starting 3 days before the i.v. injection of 500,000 B16F10 melanoma cells. The number of pulmonary metastases evaluated at day 10 after melanoma cell injection was significantly lower in laquinimod-treated mice compared to controls, which is in line with a significantly reduced lung weight (Fig. 4d). In contrast, laquinimod therapy did not decrease pulmonary metastases in AhR-deficient mice (Fig. 4e) and was also ineffective for the treatment of already established pulmonary metastases in AhR-competent mice (Fig. 4f). Collectively, these results demonstrate that laquinimod improves the effector functions of AhR-competent NK cells against B16F10 melanoma cells.

### The efficacy of laquinimod to suppress EAE is partially NK cell-dependent

**Fig. 5:**
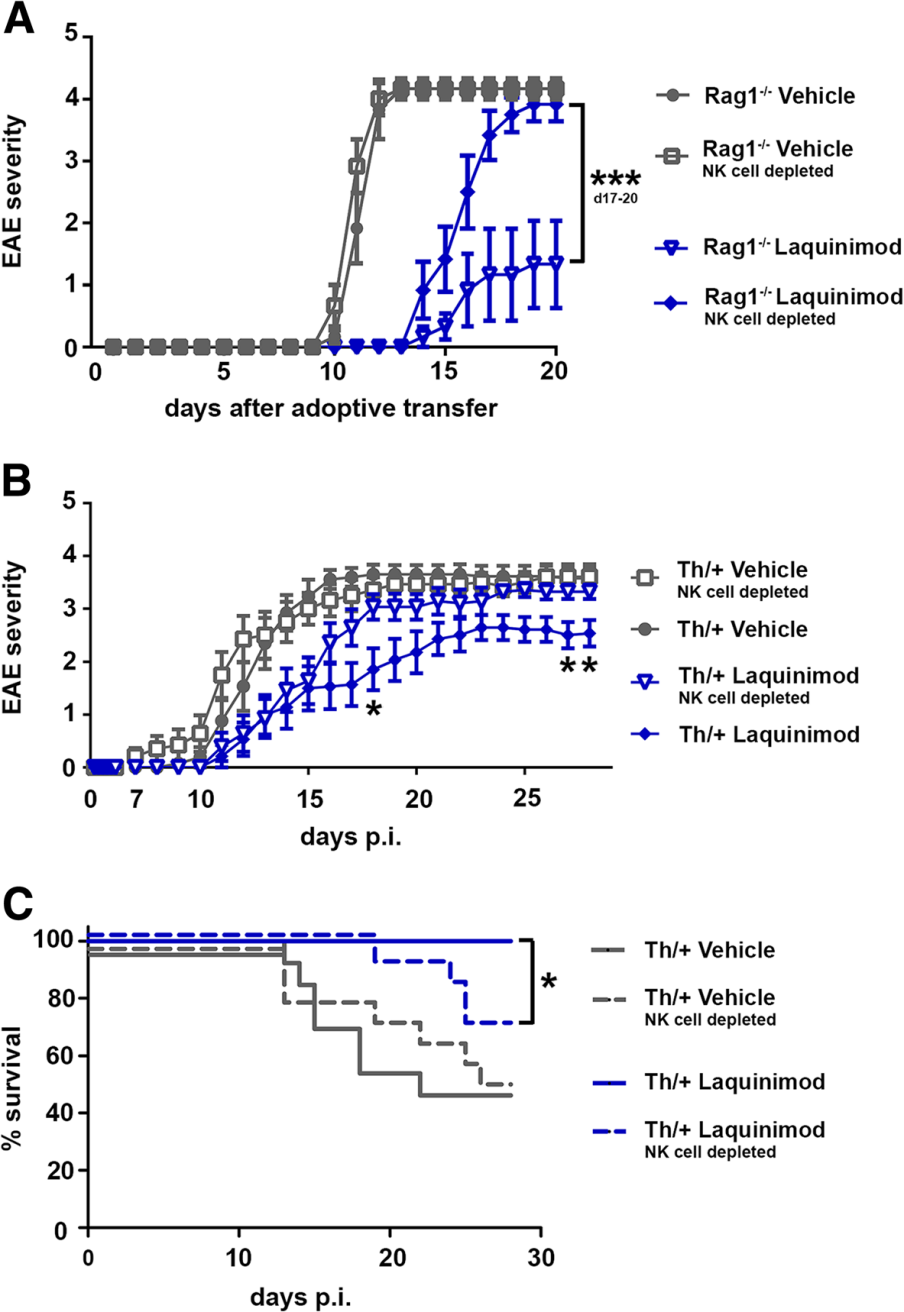
The efficacy of laquinimod to suppress EAE partially depends on NK cells. **a** EAE severity of 2D2 T cell transferred Rag1^−/−^ mice continuously depleted of NK cells by αNK1.1 (PK136) antibody application and treated with 25 mg/kg laquinimod or vehicle. Data are representative of two independent experiments and are depicted as mean ± S.E.M. *N* = 6 mice/group; ***P < 0.001, two way ANOVA. **b** EAE severity of MOG_35–55_ immunized Th/+ mice efficiently depleted of NK cells by αNK1.1 antibodies (PK136) and treated with laquinimod or vehicle for 28 days. Data are pooled from two independent experiments with and are depicted as mean ± S.E.M (*n* = 13 mice for vehicle-treated NK cell competent group, *n* = 14 mice for all other groups). *P < 0.05, between laquinimod-treated NK cell-competent or NK cell-deficient Th/+ mice, two way ANOVA. **c** Survival of EAE diseased Th/+ mice according to NK cell and treatment status. Data are pooled from two independent experiments. *P < 0.05, log-rank test

In addition to tumor cell killing, NK cells have been shown to inhibit the generation of autoreactive T cells at the time of priming in experimental models of CNS autoimmunity. To address whether the treatment efficacy of laquinimod to suppress EAE depends on the presence of NK cells, we efficiently depleted NK cells by anti-NK1.1 antibodies. To exclude any bias by the additional depletion of NK1.1 expressing NKT cells, we performed the experiments in Rag1^−/−^ mice, which lack mature T-, B-, and NKT cells. EAE was induced by the adoptive transfer of 10 Mio MOG-specific 2D2 T cells. We did not observe a clinical difference between severely affected vehicle-treated, NK cell-competent, or NK cell-deficient Rag1^−/−^ mice (Fig. 5a). Laquinimod treatment delayed the onset of EAE in both NK cell-competent or NK cell-deficient Rag1^−/−^ animals, but interestingly, EAE amelioration by laquinimod was not sustained in the absence of NK cells (Fig. 5a).

### Laquinimod is protective in immunized Th/+ mice with pathogenic MOG-specific antibodies

Activated NK cells, which are potent effector cells for antibody-dependent cellular cytotoxicity (ADCC), might be dangerous in the context of a pathogenic antibody response against myelin. We failed to detect a consistent upregulation of CD16 in laquinimod-treated animals, which is the relevant NK cell receptor for ADCC (Additional file 4: Figure S4B). To demonstrate the safety of laquinimod in MOG-specific Ig heavy-chain knock-in animals with a well-known demyelinating serum antibody response of all IgG subclasses against MOG, we treated NK cell-competent or NK cell-deficient MOG_35–55_-immunized Th/+ mice with laquinimod or vehicle. As in 2D2 T cell transferred Rag1^−/−^ mice, vehicle-treated Th/+ mice developed comparable clinical EAE irrespective of the presence or absence of NK cells (Fig. 5b). Laquinimod treatment delayed EAE development, but the sustained clinical EAE improvement and the reduction of lethality by laquinimod required the presence of NK cells (Fig. 5b, c).

### Laquinimod-derived NK cells inhibit autoreactive T cells by interacting with CD155^+^ dendritic cells

**Fig. 6:**
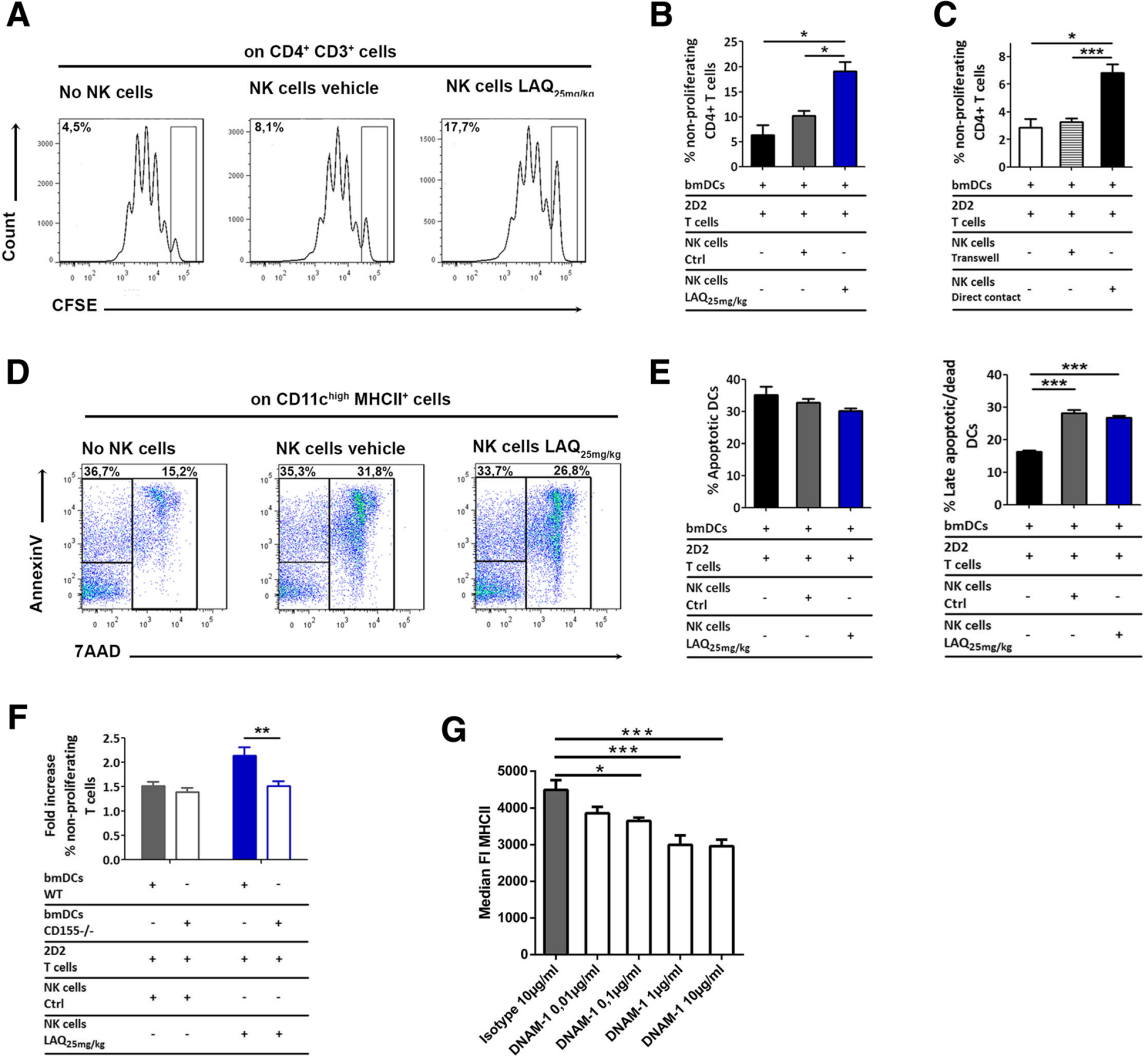
NK cells from laquinimod-treated mice inhibit autoreactive T cell proliferation by interacting with CD155^+^ DCs. **a** Representative CFSE profiles of 2D2 T cells stimulated by bmDCs and MOG_35–55_ in the presence of sorted NK cells from laquinimod- or vehicle-treated animals. Numbers in the left top quadrant indicate the percentage of non-proliferating T cells. **b** Graph quantifying the CSFE profiles of **a**. Data are presented as mean ± S.E.M. of two pooled independent experiments. *P < 0.05, Kruskal Wallis test with Dunn’s post-test. **c** Graph depicting the percentage of non-proliferating 2D2 T cells as assessed by CFSE staining if NK cells were separated by a transwell from 2D2 T cells and DCs. Data are presented as mean ± S.E.M. of three pooled independent experiments. *P < 0.05, ***P < 0.001, one-way ANOVA with Bonferroni post-test. **d** Representative flow cytometry plots showing the Annexin V/7-AAD staining of DCs in co-culture experiments with sorted splenic NK cells from laquinimod- or vehicle-treated mice, quantified in **e**. Data are presented as mean ± S.E.M. of two pooled independent experiments. ***P < 0.001, one-way ANOVA with Bonferroni post-test. **f** Quantification of the percentage of non-proliferating 2D2 T cells as assessed by CFSE labeling in co-cultures with CD155 deficient or wild-type DCs, MOG_35–55_, and splenic NK cells sorted from laquinimod- or vehicle-treated mice. Data are presented as mean ± S.E.M. and are pooled from three independent experiments. **P < 0.01, unpaired *t* test. **g** MHC class II expression (median FI) of bone marrow-derived DCs cultured in the presence of DNAM-1 Fc chimeric proteins or control IgG as assessed by flow cytometry. *P < 0.05, ***P < 0.001, one-way ANOVA with Dunnett’s post-test

Many mechanisms have been postulated by which NK cells regulate autoimmunity. At first, we examined whether NK cells can inhibit the antigen-specific proliferation of MOG-specific 2D2 T cells stimulated with bone marrow-derived dendritic cells (bmDCs) and 20 μg/ml MOG_35–55_. Ex vivo purified NK cells from laquinimod-treated animals inhibited the proliferation of 2D2 T cells better than NK cells from vehicle-treated controls (Fig. 6a, b). To clarify whether this inhibition was cell contact-dependent, we separated NK cells from 2D2 T cells and DCs by an insert. NK cells clearly did not inhibit antigen-specific T cell proliferation when they were separated from their potential targets (Fig. 6c). Next, to test whether NK cells from laquinimod-treated animals were able to suppress antigen-independent T cell proliferation, we stimulated T cells with anti-CD3/CD28. However, NK cells derived from laquinimod-treated animals failed to inhibit T cell proliferation better than NK cells from vehicle-treated controls when stimulated by antibodies instead of DCs (data not shown). In summary, the superior inhibition of T cell proliferation by ex vivo derived NK cells from laquinimod-treated animals was dependent on direct contact with DCs.

In previous studies, NK cells have been shown to kill preferentially immature DCs [42]. Indeed, the presence of NK cells significantly increased the numbers of late apoptotic/dead DCs as detected by Annexin V/7-AAD stainings in our cultures. Unexpectedly, however, NK cells from laquinimod-treated animals did not kill DCs better than NK cells from vehicle-treated controls assessed by an Annexin V/7AAD (Fig. 6d, e) or by a crystal violet assay (Additional file 4: Figure S4C). To find out which interaction between NK cells from laquinimod-treated mice and DCs impairs T cell proliferation, we generated bmDCs from CD155-deficient animals. Of note, NK cells from laquinimod-treated mice failed to suppress T cell proliferation better than NK cells from vehicle-treated animals when DCs were deficient of CD155 (Fig. 6f). CD4 T cell proliferation depends on antigen presentation by MHC class II and co-stimulatory signals which can be delivered by CD40, CD80, or CD86. In further experiments, we replaced NK cells by recombinant DNAM-1 and analyzed the expression levels of MHCII, CD40, CD80, and CD86 on bmDCs. DNAM-1 downregulated MHCII expression in a dose-dependent manner (Fig. 6g), whereas co-stimulatory molecules remained unchanged (Additional file 5: Figure S5A-C).

In summary, our results indicate that the AhR-dependent activation of NK cells might be utilized to suppress CNS autoimmunity by decreasing the immunogenicity of CD155^+^ DCs.

## Discussion

The bidirectional crosstalk between DCs and NK cells can influence the development of adaptive immune responses, thus providing exciting possibilities for the treatment of autoimmune diseases. Laquinimod, a prototypic quinoline-3-carboxamide, modulates important functions of both innate immune cell players. It reduces CD11c^high^ MHCII^+^ DCs [29], which has been addressed in previous studies and activates NK cells. Both responses depend on the presence of the AhR. In the present study, we report that the AhR-mediated activation of NK cells augments anti-tumor immunity and is relevant for maintaining the efficacy of laquinimod to ameliorate CNS autoimmunity.

Laquinimod’s activating effect on NK cells and down-modulatory effect on DCs is compatible with an AhR agonistic activity [39], and recent studies have shown that the AhR is involved in NK cell activation in mice and humans. NK cells from AhR-deficient mice had poorer cytotoxic activity against RMA-S lymphoma cells as compared to NK cells from wild-type mice [39], treating animals with the potent AhR ligand 6-formylindolo (3,2-b) carbazole (FICZ) enhanced NK cell control of RMA-S tumors. DNAM-1, which is consistently upregulated on AhR-competent NK cells by laquinimod, is an adhesion molecule, originally shown to influence NK and T cell cytotoxicity upon interaction with its ligands, CD112 and CD155 [43]. In this regard, DNAM-1 appears to be relevant for the control of poorly immunogenic B16F10 melanoma metastases [5, 44]. Correspondingly, laquinimod-derived NK cells killed in vitro B16F10 melanoma cells better than NK cells from vehicle-treated controls*.* Likewise, they reduced the number of pulmonary metastases in vivo, albeit only when the treatment started at incipient stages. Our results are in line with the current paradigm that increasing NK cell activity is preferentially beneficial against circulating tumor cells but less efficient once the tumor cells have extravasated and formed solid tumors [45].

Of note, besides shifting the NK cell subset distribution towards CD27^+^ NK cells, laquinimod activates and improves the effector functions of both CD27 and CD11b single-positive NK cells. To strengthen effector functions of CD27, single-positive NK cells might be particularly relevant for MS, since CD27 single-positive NK cells and their human CD56^bright^ NK cell counterparts are the dominant intrathecal and lymph node NK cell population [46]. Furthermore, a number of studies suggested that not only the immune-regulatory functions of NK cells might be impaired in MS but also their differentiation from the more immature CD56^bright^ to the more mature CD56^dim^ stage.

Recent data have expanded our knowledge on the function of DNAM-1 in NK cell biology and demonstrate its relevance for immune synapse formation, stable target cell conjugates, NK cell education, and memory differentiation [47–49]. In addition, DNAM-1 expression separates NK cells into two functional subsets [41], with DNAM-1^+^ NK cells producing higher amounts of cytokines, having better proliferation capabilities and providing better control of lung melanoma metastases compared to DNAM-1^−^ NK cells. Interestingly, DNAM-1 receptor expression on NK cells is decreased in both cancer and MS patients and associated with impaired NK cell functions [50]. Therefore, increasing the number of DNAM-1^+^ NK cells might not only have therapeutic implications in a variety of malignant human tumors, expressing DNAM-1 ligands [51, 52], but also for restoring the NK cell-mediated control in autoimmune diseases.

The contribution of NK cells in MS pathogenesis still remains controversial, but temporal correlations between reduced NK cell numbers with decreased cytotoxicity and MS relapses have been established. These correlations suggest that NK cells may play a regulatory role in MS by a number of possible mechanisms: First, NK cells can inhibit cytokine secretion or kill T cells in a Fas-dependent or granzyme-dependent manner [13, 53, 54]. Second, DNAM-1 CD155 interactions between NK cell and activated T cells might contribute to T cell cytolysis [10]. Third, NK cells can ameliorate Th17-driven autoimmune responses by interacting with DCs through IFNγ and IL-27, which directs Tr1 T cell differentiation [55] or by interacting with microglia cells within the CNS [23]. Fourth, our data uncover an additional mechanism underlying how NK cells might inhibit autoreactive T cells, which requires cell-to-cell contact with CD155^+^ DCs to suppress antigen-specific T cell proliferation. This interaction might impair antigen presentation by DCs, since DNAM-1 dose-dependently downregulates MHC class II expression without changing the expression of co-stimulatory molecules. The clinical relevance of this interaction is underscored by the impaired sustainability of laquinimod to suppress CNS autoimmunity in the absence of NK cells.

ADCC effector functions of NK cells are important for the therapeutic efficacy of monoclonal antibodies in treating human disorders [56]. NK cells also lysed oligodendrocytes or MOG-transfected tumor cells in vitro in the presence of MOG-specific, patient-derived serum antibodies. We therefore analyzed whether NK cell activation is safe in the setting of pathogenic MOG-specific antibodies. We did not observe a clinical difference in Th/+ mice with or without NK cells, and laquinimod is more effectively protective in NK cell-competent than NK cell-deficient mice. Accordingly, the major receptor for ADCC in NK cells remains unchanged in response to laquinimod therapy.

## Conclusions

In summary, we demonstrate that the NK cell-activating functions of the AhR can be used to suppress CNS autoimmunity and improve anti-tumor immunity. We show that the quinoline-3-carboxamide laquinimod improves the immunoregulatory properties of NK cells by upregulating their DNAX accessory molecule 1 (DNAM-1) cell surface expression. The enhanced NK cell-mediated inhibition of autoreactive T cells requires direct cell contact of NK cells with CD155^+^ dendritic cells (DCs), thereby impairing MHC-II antigen presentation. Our findings highlight the importance of the immunoregulatory DNAM-1/CD155 network between NK cells and DCs, an interaction that can be exploited to treat CNS autoimmune diseases. Furthermore, our results suggest that increasing the availability of natural AhR ligands might be of therapeutic potential for autoimmune and neoplastic diseases.

## Additional files

Additional file 1: **Figure S1.** Graphs depicting the MFI of DNAM-1 (A) or TIGIT (B) on splenic CD3+ T cells 11 days after MOG_35-55_ immunization and oral treatment with 25 mg/kg laquinimod or vehicle. Data are representative of two independent experiments and presented as mean ± S.E.M.. **P < 0.01, unpaired t test. (JPG 47 kb)

Additional file 2: **Figure S2.** (A) Flow cytometry analysis of splenic DCs in Itgax DTR mice depleted by 100 ng DTx (right dot blot) or vehicle (left dot blot) on day 3 after injection. Representative graph depicting the number of DCs/105 leukocytes in the spleens of DTx or vehicle-injected animals on day 3 after injection. ***P < 0.001, unpaired *t* test. (B) Flow cytometry analysis of NK cell numbers in the blood of aNK1.1 (300 μg PK136 every other day) or isotype control ab treated mice prior to MOG_35–55_ immunization and its quantification. ***P < 0.001, unpaired *t* test. (JPG 296 kb)

Additional file 3: **Figure S3.** (A) Representative flow cytometry of splenic NK1.1/AhR expression in MOG_35–55_ immunized mice. (B) Representative flow cytometry analysis of NK1.1/ CD69 expression in naïve C57BL/6 or AhR−/− mice treated for 11 days with 25 mg/kg laquinimod or vehicle. (C) Representative flow cytometry analysis of DCs in the spleens of C57BL/6 and AhR-deficient mice treated with 25 mg/kg laquinimod or vehicle for 11 days. ***P < 0.001, two-way ANOVA. (TIF 1044 kb)

Additional file 4: **Figure S4.** (A) Representative flow cytometry analysis of CD112/CD155 expression on B16F10 melanoma cells. (B) Graph depicting the CD16 expression (MFI) on splenic NK cells as assessed by flow cytometry 11 days after laquinimod treatment. *P* > 0.05, unpaired *t* test. (C) Crystal violet assay graph depicting the survival of DCs in coculture experiments with NK cells sorted from laquinimod- or vehicle-treated mice. Data are presented as mean ± S.E.M. *P* > 0.05, unpaired *t* test. (JPG 107 kb)

Additional file 5: **Figure S5.** Expression (median FI) of CD40 (A), CD80 (B), and CD86 (C) on bone marrow-derived DCs cultivated in the presence of 1 ng/ml LPS and various concentrations of DNAM-1 Fc chimeric protein for 24 h. Representative experiment out of two performed. Data are presented as mean ± S.E.M. *P* > 0.05, one-way ANOVA with Dunnett’s post-test. (JPG 149 kb)

## Abbreviations

AhR: Aryl hydrocarbon receptor
bm DCs: Bone marrow-derived DCs
CD112: Cluster of differentiation 112
CD155: Cluster of differentiation 155
CNS: Central nervous system
DC: Dendritic cells
DNAM1: DNAX accessory molecule 1
EAE: Experimental autoimmune encephalomyelitis
FACS: Fluorescence-activated cell sorting
IFNγ: Interferon gamma
MACS: Magnetic-activated cell sorting
MOG: Myelin oligodendrocyte glycoprotein
MS: Multiple sclerosis
NK: Natural killer
