# Supplementary figures and images for: Laquinimod, a prototypic quinoline-3-carboxamide and aryl hydrocarbon receptor agonist, utilizes a CD155-mediated natural killer/dendritic cell interaction to suppress CNS autoimmunity
Source: J Neuroinflammation. 2019 Feb 26;16:49. doi: 10.1186/s12974-019-1437-0 (PMC6390632; doi:10.1186/s12974-019-1437-0)

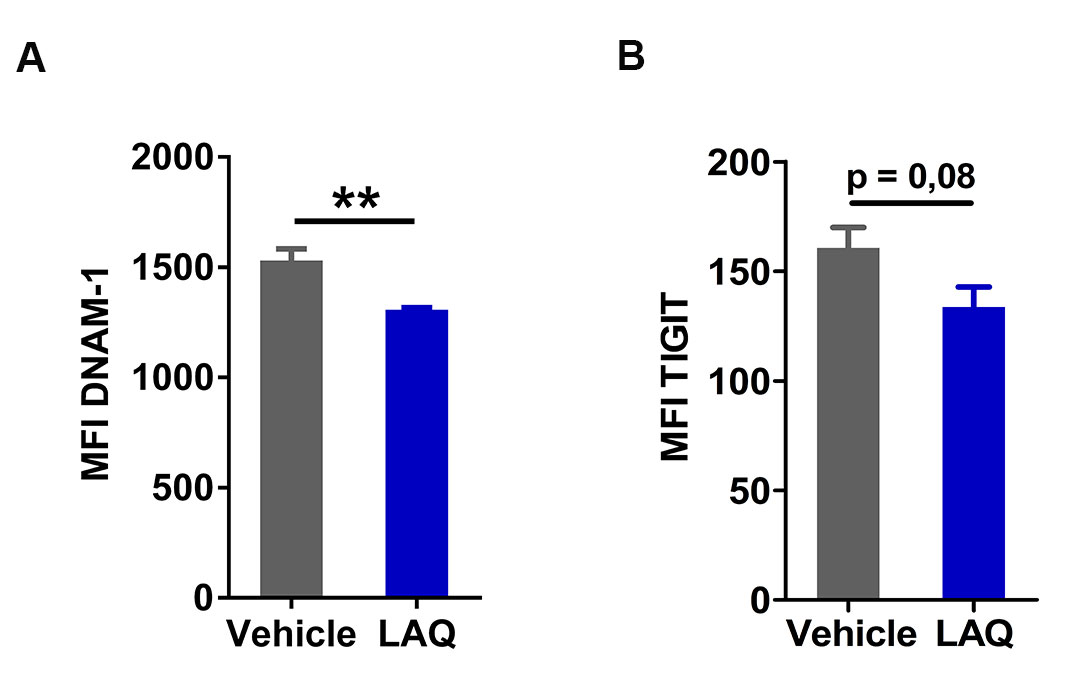

Supplement: Supplementary file 1 — Figure S1. Graphs depicting the MFI of DNAM-1 (A) or TIGIT (B) on splenic CD3+ T cells 11 days after MOG35-55 immunization and oral treatment with 25 mg/kg laquinimod or vehicle. Data are representative of two independent experiments and presented as mean ± S.E.M.. **P < 0.01, unpaired t test. (JPG 47 kb) [file 12974_2019_1437_MOESM1_ESM.jpg]

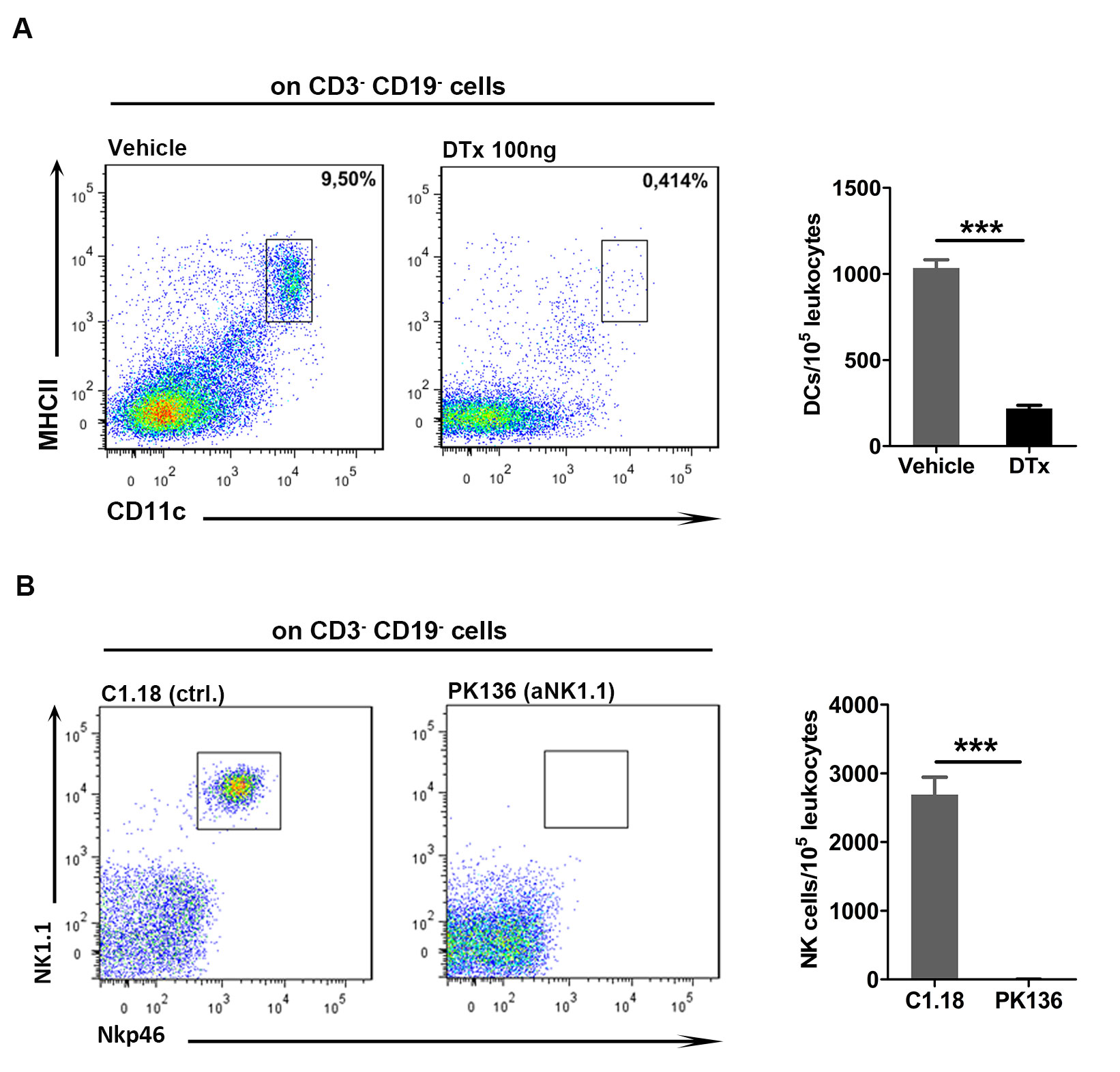

Supplement: Supplementary file 2 — Figure S2. (A) Flow cytometry analysis of splenic DCs in Itgax DTR mice depleted by 100 ng DTx (right dot blot) or vehicle (left dot blot) on day 3 after injection. Representative graph depicting the number of DCs/105 leukocytes in the spleens of DTx or vehicle-injected animals on day 3 after injection. ***P < 0.001, unpaired t test. (B) Flow cytometry analysis of NK cell numbers in the blood of aNK1.1 (300 μg PK136 every other day) or isotype control ab treated mice prior to MOG35–55 immunization and its quantification. ***P < 0.001, unpaired t test. (JPG 296 kb) [file 12974_2019_1437_MOESM2_ESM.jpg]

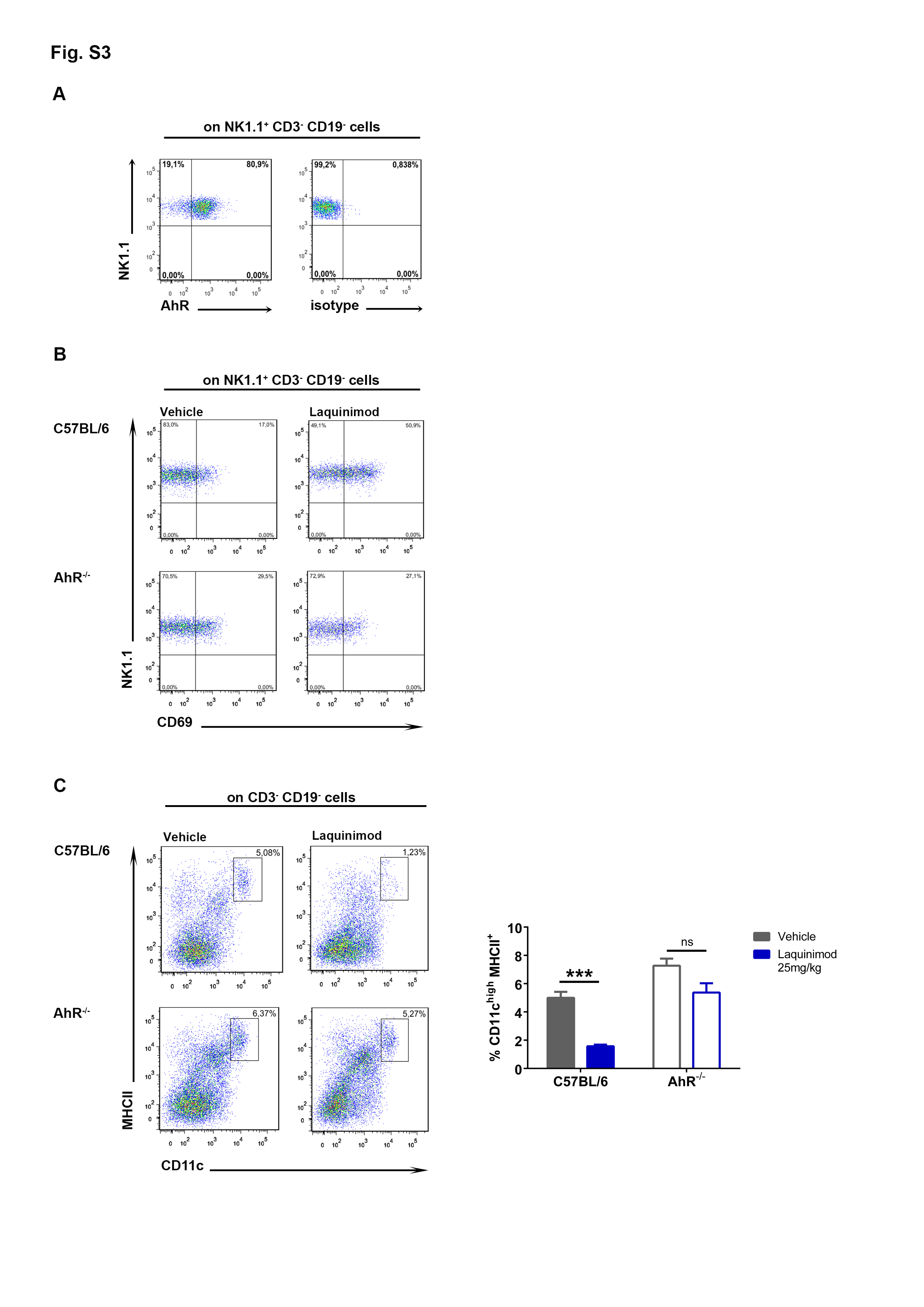

Supplement: Supplementary file 3 — Figure S3. (A) Representative flow cytometry of splenic NK1.1/AhR expression in MOG35–55 immunized mice. (B) Representative flow cytometry analysis of NK1.1/ CD69 expression in naïve C57BL/6 or AhR−/− mice treated for 11 days with 25 mg/kg laquinimod or vehicle. (C) Representative flow cytometry analysis of DCs in the spleens of C57BL/6 and AhR-deficient mice treated with 25 mg/kg laquinimod or vehicle for 11 days. ***P < 0.001, two-way ANOVA. (TIF 1044 kb) [file 12974_2019_1437_MOESM3_ESM.tif]

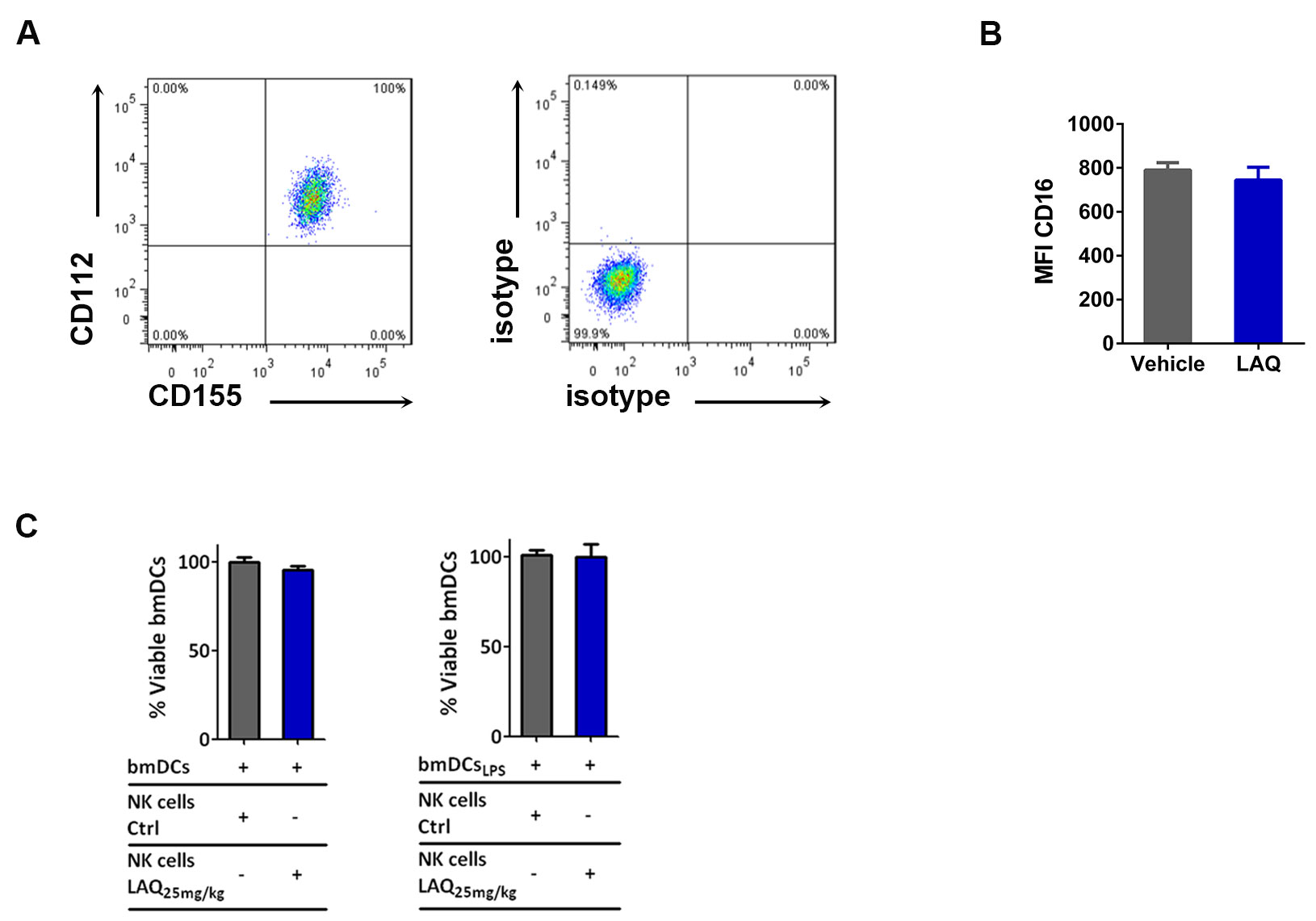

Supplement: Supplementary file 4 — Figure S4. (A) Representative flow cytometry analysis of CD112/CD155 expression on B16F10 melanoma cells. (B) Graph depicting the CD16 expression (MFI) on splenic NK cells as assessed by flow cytometry 11 days after laquinimod treatment. P > 0.05, unpaired t test. (C) Crystal violet assay graph depicting the survival of DCs in coculture experiments with NK cells sorted from laquinimod- or vehicle-treated mice. Data are presented as mean ± S.E.M. P > 0.05, unpaired t test. (JPG 107 kb) [file 12974_2019_1437_MOESM4_ESM.jpg]

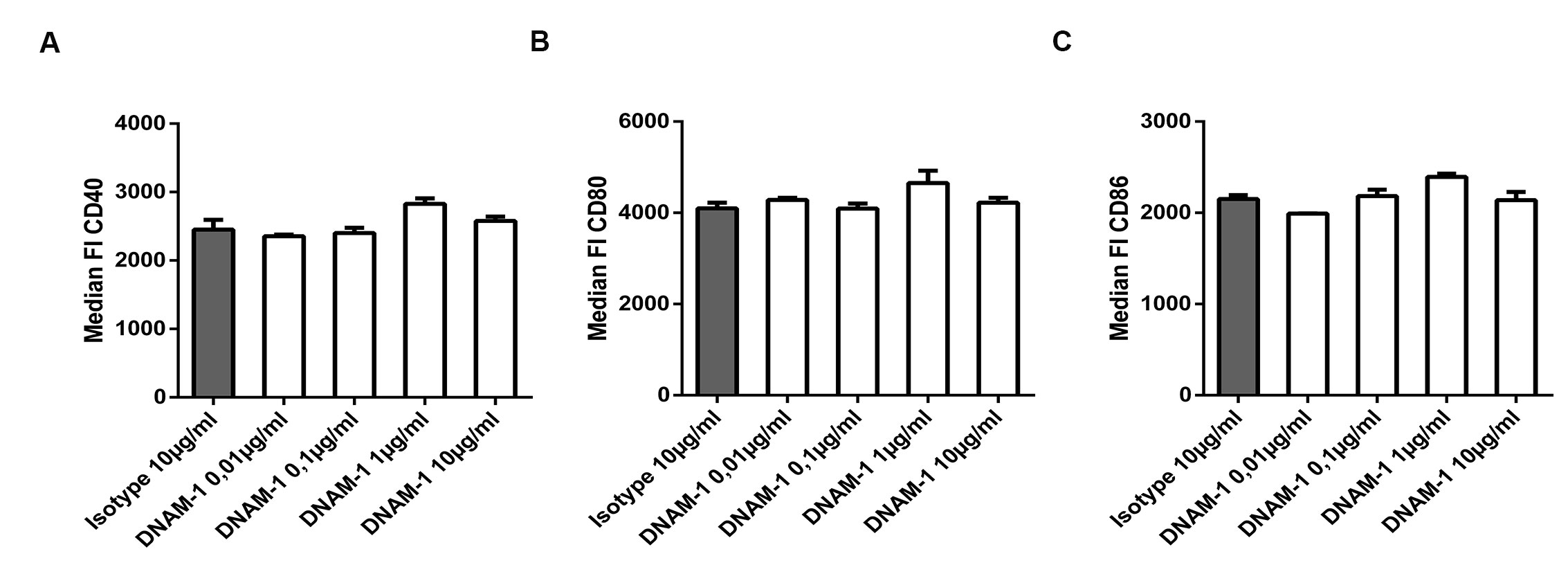

Supplement: Supplementary file 5 — Figure S5. Expression (median FI) of CD40 (A), CD80 (B), and CD86 (C) on bone marrow-derived DCs cultivated in the presence of 1 ng/ml LPS and various concentrations of DNAM-1 Fc chimeric protein for 24 h. Representative experiment out of two performed. Data are presented as mean ± S.E.M. P > 0.05, one-way ANOVA with Dunnett’s post-test. (JPG 149 kb) [file 12974_2019_1437_MOESM5_ESM.jpg]
